# Premature aging in mice with error-prone protein synthesis

**DOI:** 10.1126/sciadv.abl9051

**Published:** 2022-03-02

**Authors:** Dimitri Shcherbakov, Martina Nigri, Rashid Akbergenov, Margarita Brilkova, Matilde Mantovani, Patricia Isnard Petit, Amandine Grimm, Agnieszka A. Karol, Youjin Teo, Adrián Cortés Sanchón, Yadhu Kumar, Anne Eckert, Kader Thiam, Petra Seebeck, David P. Wolfer, Erik C. Böttger

**Affiliations:** 1 Institut für Medizinische Mikrobiologie, Universität Zürich, CH-8006 Zurich, Switzerland.; 2 Anatomisches Institut, Universität Zürich, and Institut für Bewegungswissenschaften und Sport, ETH Zürich, CH-8057 Zurich, Switzerland.; 3 genOway, F-69362 Lyon Cedex 07, France.; 4 Universitäre Psychiatrische Kliniken Basel, Transfaculty Research Platform Molecular and Cognitive Neurosciences, CH-4055 Basel, Switzerland.; 5 Musculoskeletal Research Unit (MSRU), Vetsuisse Faculty, University of Zurich, CH-8057 Zurich, Switzerland.; 6 Eurofins Genomics Europe Sequencing GmbH, D-78467 Konstanz, Germany.; 7 Zurich Integrative Rodent Physiology (ZIRP), University of Zurich, CH-8057 Zurich, Switzerland.

## Abstract

The main source of error in gene expression is messenger RNA decoding by the ribosome. Translational accuracy has been suggested on a purely correlative basis to positively coincide with maximum possible life span among different rodent species, but causal evidence that translation errors accelerate aging in vivo and limit life span is lacking. We have now addressed this question experimentally by creating heterozygous knock-in mice that express the ribosomal ambiguity mutation RPS9 D95N, resulting in genome-wide error-prone translation. Here, we show that *Rps9* D95N knock-in mice exhibit reduced life span and a premature onset of numerous aging-related phenotypes, such as reduced weight, chest deformation, hunchback posture, poor fur condition, and urinary syndrome, together with lymphopenia, increased levels of reactive oxygen species–inflicted damage, accelerated age-related changes in DNA methylation, and telomere attrition. Our results provide an experimental link between translational accuracy, life span, and aging-related phenotypes in mammals.

## Teaser

Decreased translational accuracy shortens life span and accelerates the onset of age-related phenotypes in mammals.

## INTRODUCTION

The accuracy of gene expression is central in translating genomic information into functional proteins. However, gene expression is an error-prone process. The main contribution to errors in gene expression comes from mRNA decoding by the ribosome with an average error rate of 10^−4^, compared to error frequencies of 10^−8^ in DNA replication and 10^−6^ in RNA transcription (*1*, *2*). Translational accuracy has been suggested to positively correlate with maximum possible life span among different rodent species, pointing to a possible link between translation accuracy, life span, and potentially age-accelerating effects of translational errors (*3*, *4*). In addition, reductions in protein synthesis fidelity have recently been connected to malignant disease and oncogene-mediated tumor development (*5*). The idea that aging and age-related diseases may be related to the accuracy of protein synthesis evolved some 60 years ago, resulting in the error catastrophe theory of aging (*6*, *7*). While this theory has been refuted, mainly based on theoretical considerations and studies in unicellular prokaryotes (*8*, *9*), evidence has been provided that aging of mammalian cells in cell culture may be accompanied by an age-related increase in translational error frequency (*10*, *11*).

The intrinsic error in protein synthesis together with the requirement to maintain a proper proteome makes proteostasis a major concern over a long life span (*12*, *13*). The stability of the proteome is constantly challenged by protein misfolding, often due to genetic polymorphisms, and errors during protein translation (*14*). Constant surveillance by an integrated network of chaperones and protein degradation machineries is therefore required to maintain cellular proteostasis (*13*, *15*, *16*). A decline in proteostasis capacity has been reported to be an early event in aging and an inherent part of the aging process in various organisms (*17*–*19*).

To directly test the possibility that translational accuracy contributes to aging, we decided to develop an experimental mammalian model that displays genome-wide error-prone translation. To alter translation accuracy, we chose a genetic approach based on the identification of a ribosomal ambiguity mutation (*ram*) that confers error-prone translation in mammalian protein synthesis. *Rams* are a class of mutations that increase the natural error rate of translation in a random manner by interfering with the initial phase of tRNA selection resulting in reduced discrimination against near-cognate aminoacyl-tRNAs (aa-tRNAs) (*20*–*22*). This mimics the mechanism of inherent errors in protein synthesis, which largely result from incorporation of mismatched near-cognate aa-tRNAs during mRNA decoding (*1*, *23*). We have previously reported on RPS2-A226Y as the first *ram* characterized in higher eukaryotes (*24*). However, attempts to stably introduce this mutation into the mouse genome have failed and resulted in unwanted genetic mosaicism (*25*).

To generate a genetic model with unbiased, tissue-independent, and genome-wide expression of error-prone translation, we built on RPS9-D94N, a well-known *ram* in the lower eukaryote *Saccharomyces cerevisiae* (*26*, *27*). Following demonstration that the mammalian homolog of yeast RPS9-D94N, i.e., RPS9-D95N, confers error-prone translation on mammalian ribosomes, we subsequently introduced this mutation into the genome of mice, resulting in knock-in mice that express the RPS9-D95N mutation in a genetically stable, heterozygous, and organism-wide manner. *Rps9* D95N mice exhibited reduced life span and premature onset of numerous age-associated phenotypic alterations, thus providing an experimental link between translational accuracy, life span, and aging-related phenotypes in mammals.

## RESULTS

### RPS9-D95N is a ribosomal ambiguity mutation in higher eukaryotes

By aligning the RPS9 (uS4) sequences of *S. cerevisiae*, *Mus musculus*, and *Homo sapiens*, we identified D95N as the homologous mutation in mouse and human RPS9 corresponding to D94N in yeast (fig. S1). Structural modeling suggests that in yeast the RPS9-D94N mutation affects the RPS9(uS4)-RPS2(uS5) interface as it results in disruption of polar contacts between the aspartic acid at position 94 of RPS9 and the arginine residue at position 174 of RPS2 (fig. S2, A and B). Similarly, the RPS9-D95N mutation in mammalian ribosomes would disrupt the polar contacts between the aspartic acid at position 95 of RPS9 and the lysine residue at position 173 of RPS2 (fig. S2, C and D). Weakening of contacts at the ribosomal uS4-uS5 interface favors the domain closure process of the small ribosomal subunit, which is thought to stabilize ternary complex binding to the ribosome, facilitating incorporation of mismatched near-cognate aa-tRNAs during mRNA decoding (*2*, *22*). On the basis of the modeling results, we hypothesized that RPS9 D95N would confer mistranslation in higher eukaryotes.

**Fig. 1.**
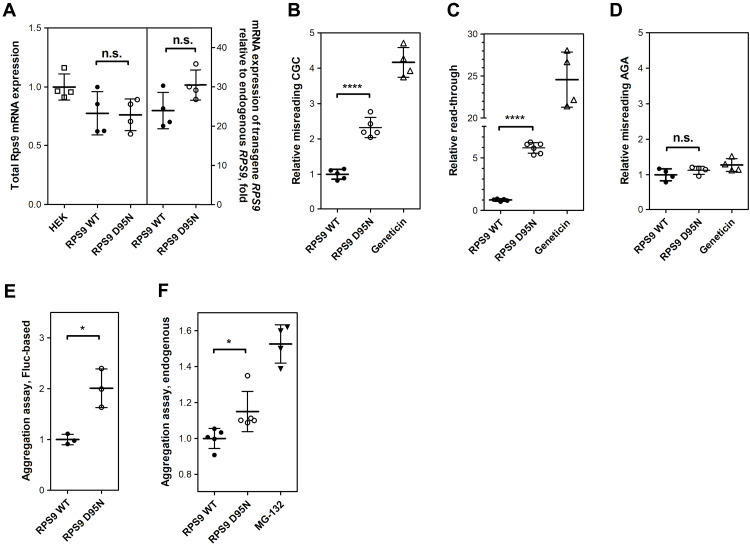
Characterization of *Rps9* D95N mutant cells. (**A**) *Rps9* mRNA expression measured using TaqMan qRT-PCR. Data shown represent the average of four independent experiments (*N* = 4 clones each for *RPS9* WT and D95N; ± SEM). Left, total expression of *Rps9* mRNA in *Rps9*-transfected cells compared to untransfected HEK WT; right, relative expression of transgenic *Rps9* mRNA in comparison to endogenous *Rps9* mRNA in *Rps9*-transfected cells, fold change. (**B** to **D**) Mistranslation assays using dual-luciferase system. (B) Misincorporation of near-cognate aa-tRNA, (C) read-through, and (D) misincorporation of noncognate aa-tRNA. Results are derived from the ratio hFluc/hRluc, given in fold induction. *Rps9* WT cells are set as 1 (*N* = 6 clones each for *Rps9* WT and D95N; ± SEM); geneticin is used as a positive control. (**E**) Firefly luciferase–based protein aggregation assay. The data shown represent the quantification of band intensities from three independent experiments (*N* = 3 clones each for *Rps9* WT and D95N; ± SEM). A representative Western blot can be found in fig. S3G. (**F**) Aggregation of endogenous cellular proteins assessed by a PROTEOSTAT aggresome detection kit (*N* = 5 clones each for *Rps9* WT and D95N; ± SEM). Treatment with the proteasome inhibitor MG-132 was used as a positive control. *P < 0.05 and ****P < 0.0001.

To experimentally assess whether D95N confers mistranslation in mammalian ribosomes, we generated stably transfected human embryonic kidney (HEK) 293 cells constitutively expressing wild-type (WT) RPS9 or mutant RPS9-D95N. Cells transfected with WT *Rps9* or mutant *Rps9* D95N are subsequently termed RPS9 WT or RPS9-D95N, compared to untransfected HEK293 cells, termed HEK293. Transfection with myc-tagged WT *Rps9* and mutant *Rps9* D95N demonstrated that the transfected RPS9 protein is incorporated into actively translating polyribosomes (fig. S3, A and B). The expression level of the *Rps9* transgene was first estimated by expression of the internal ribosomal entry site (IRES)–green fluorescent protein (GFP) reporter fused downstream of transgenic *Rps9*. No difference in GFP expression was found in cells transfected with *Rps9* WT or *Rps9* D95N mutant (fig. S3C). To more precisely study transgenic versus endogenous *Rps9* expression and to assess levels of total *Rps9* mRNA, we performed TaqMan quantitative reverse transcription polymerase chain reaction (qRT-PCR). While total amounts of *Rps9* mRNA were similar in untransfected HEK293 cells and HEK293 cells transfected with *Rps9* WT or *Rps9* D95N, the relative mRNA levels for transgenic *Rps9* mRNA were 25- to 30-fold that of endogenous *Rps9* mRNA, with no significant difference between transgenic WT and transgenic D95N mutant (Fig. 1A). The high expression of transgenic *Rps9* mRNA as compared to endogenous *Rps9* mRNA probably reflects a known autoregulatory circuit of *Rps9* expression, with degradation of endogenous *Rps9* mRNA occurring by nonsense-mediated decay, induced by the expression of transgenic *Rps9* (*28*). As a result, while total amounts of *Rps9* mRNA are similar in nontransfected and transfected HEK293 cells, more than 90% of *Rps9* mRNA in the transfected cells is transgenic *Rps9* mRNA.

The D95N mutation did not measurably affect translational activity as assessed by ^35^S-methionine incorporation, measurements of translational elongation by polysome run-off assays, or polysome profiling (fig. S3, D to F). We used dual-luciferase reporters to assess mutant RPS9-mediated misreading and read-through (*24*). In this assay, the active site H245 of firefly luciferase (Fluc) was mutated from CAC to near-cognate CGC, resulting in a nonfunctional protein. To measure read-through, a stop codon TGA was introduced at D357 of Fluc to produce a truncated and nonactive Fluc. The misreading agent geneticin was included as a positive control for both reporters. Compared to RPS9 WT, we found that RPS9-D95N mutant cells showed increased levels of near-cognate misreading (2.3-fold) and stop codon read-through (6.0-fold) (Fig. 1, B and C). As is typical for *rams*, *Rps9* D95N affects misreading of near-cognate, but not noncognate, codons (Fig. 1D). To determine whether D95N-induced mistranslation resulted in increased protein aggregation, we used the misfolding-prone protein firefly luciferase (*29*) as reporter for transfection of cells and analyzed the soluble and pellet fraction of cellular extracts by Western blotting using antibodies against Fluc. The misreading mutant D95N showed higher levels of luciferase aggregation compared to RPS9 WT, as indicated by the shift in Fluc distribution toward the insoluble pellet fraction (Fig. 1E and fig. S3G). We also assessed aggregation of endogenous proteins in RPS9-D95N mutant cells versus RPS9 WT transfected cells using a PROTEOSTAT aggresome detection kit (Enzo Life Sciences). Aggregation of endogenous proteins was increased in the RPS9-D95N mutant cells in comparison to RPS9 WT cells (Fig. 1F).

### Generation of *Rps9* D95N heterozygous knock-in mice

To assess the consequences of D95N-induced mistranslation in vivo, we generated heterozygous *Rps9* D95N mutant mice using a knock-in strategy (see Materials and Methods for further details and fig. S4). The knock-in was performed at the mouse *Rps9* locus, i.e., the mutant *Rps9* gene expression is under control of its endogenous promoter, and the insertion point was selected not to disrupt any potential regulatory region identified by consensus sequences. We first generated the *Rps9*^D95NNeo^ allele in embryonic stem (ES) cells and obtained germline transmission. Next, we excised the neomycin resistance gene by mating male chimeras with C57BL/6 Cre deleter female mice, thus generating heterozygous *Rps9*^D95N/WT^ mice devoid of the neomycin cassette. Expression of *Rps9* D95N was assessed by RT-PCR and sequencing. Both WT and mutant *Rps9* transcripts were detected [570 base pairs (bp)] in tissues of 6- to 7-week-old animals. The presence of the D95N mutation was confirmed by RT-PCR sequencing and indicated equivalent amounts of WT *Rps9* and mutant *Rps9* D95N mutant mRNA in the heterozygous D95N animals (fig. S4). Heterozygous *Rps9* D95N mutant mice were viable and fertile, while the presence of the mutation on both alleles in homozygous mice was embryonic lethal.

We next wished to demonstrate that the *Rps9* D95N mutation impairs translational fidelity in transgenic mouse tissue. To directly assess translational fidelity in animal tissue, we extracted ribosomes from liver of heterozygous D95N animals and WT littermate controls for in vitro translation assays. As a common type of translational error, we measured stop codon read-through using dual-luciferase reporters. Compared to liver ribosomes from WT littermate controls, liver ribosomes from heterozygous D95N animals showed significantly increased levels of stop codon read-through (fig. S4F).

### *Rps9* D95N mice show premature onset of age-related phenotypes

**Fig. 2.**
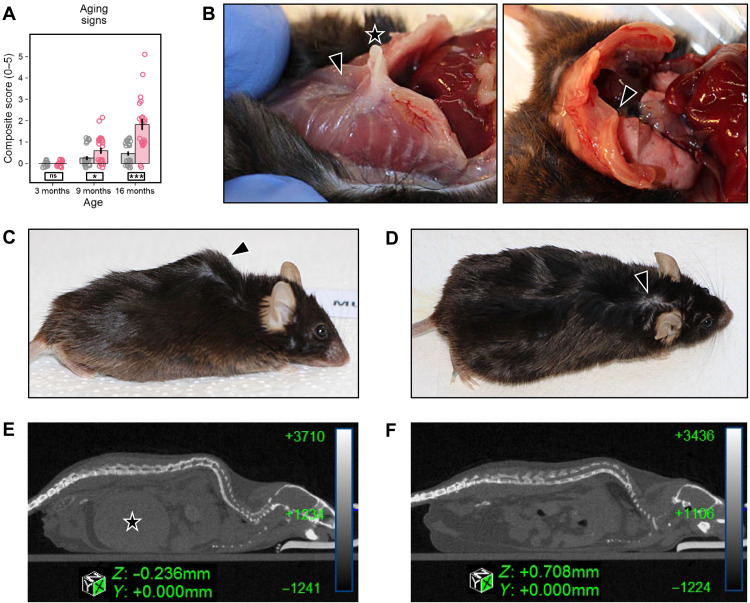
Signs of premature aging in *Rps9* D95N mice. (**A**) Composite score reflecting the presence or absence of five aging signs: chest deformation, hunchback posture, altered fur (alopecia, depigmentation, and scruffiness), eye problems (cataract and inflammation), and poor body condition [genotype: *F*_1,116_ = 27.89, *P* < 0.0001, η^2^ = 0.19; age: *F*_1,116_ = 59.12, *P* < 0.0001, η^2^ = 0.34; genotype × age: *F*_1,116_ = 23.16, *P* < 0.0001, η^2^ = 0.17; genotype × sex: *F*_1,116_ = 3.504, *P* = 0.0637, η^2^ = 0.03; genotype × sex × age: *F*_1,116_ = 1.363, not significant (ns)]. Red, *Rps9* D95N mutants; gray, WT. Mean, SE, and individual data points plotted with vertical and horizontal jittering. Genotype split post hoc by age: ***P < 0.001, *P < 0.05, ns *P* ≥ 0.1. Number of animals: 3-month WT, 8 F/8 M; *Rps9* D95N, 8 F/8 M; 9-month WT, 12 F/12 M; *Rps9* D95N, 10 F/12 M; 16-month WT, 12 F/12 M; *Rps9* D95N, 9 F/13 M. (**B**) Chest deformities were observed only in the oldest age cohort (1 of 24 WT, 16 of 22 *Rps9* D95N; χ^2^ test, *P* < 0.0001). Left, indented sternum (arrowhead) with protruding xiphoid (star); right, asymmetrical chest with deformed ribs (arrowhead). (**C**) *Rps9* D95N mouse showing typical hunchback posture. (**D**) Poor fur condition with bald patches in a *Rps9* D95N mouse (arrowhead). (**E** and **F**) Mouse urinary syndrome: micro-CT images of a 17-month male *Rps9* D95N mutant (E) and a 17-month male WT mouse (F). The *Rps9* D95N mouse shows a massively filled bladder (star), while the bladder is empty and thus invisible in the WT mouse.

Young *Rps9* D95N mice were indistinguishable from WT littermates, but long-term follow-up revealed a notable premature aging phenotype beginning at about 9 months of age, consisting of hair loss, graying, kyphosis, chest deformation, hunchback postures, eye cataracts, and poor body condition (Fig. 2, A to D). These clinical symptoms of aging are common in mice and were occasionally observed in control mice of our study. Among *Rps9* D95N mice, not only there were more individuals showing signs of aging but also the number of aging signs present was increased. While at 16 months only about half of the control mice showed at maximum one symptom of aging, almost all *Rps9* D95N mice were affected, many of them showing multiple age-related symptoms. Quantification of aging symptoms using a composite score revealed a highly significant effect of the *Rps9* D95N mutation (Fig. 2A). In addition, at age 18 months, 8 of 10 male *Rps9* D95N mutants but 0 of 15 male controls (χ^2^ test, *P* < 0.0001) suffered from obstructive uropathy (Fig. 2, E and F). Obstructive uropathy due to blockage of the lower urinary tract is frequently observed in male C57BL/6 mice >24 months of age (*30*, *31*).

### Reduced life span, altered body composition, and deficient motor function in *Rps9* D95N mice

**Fig. 3.**
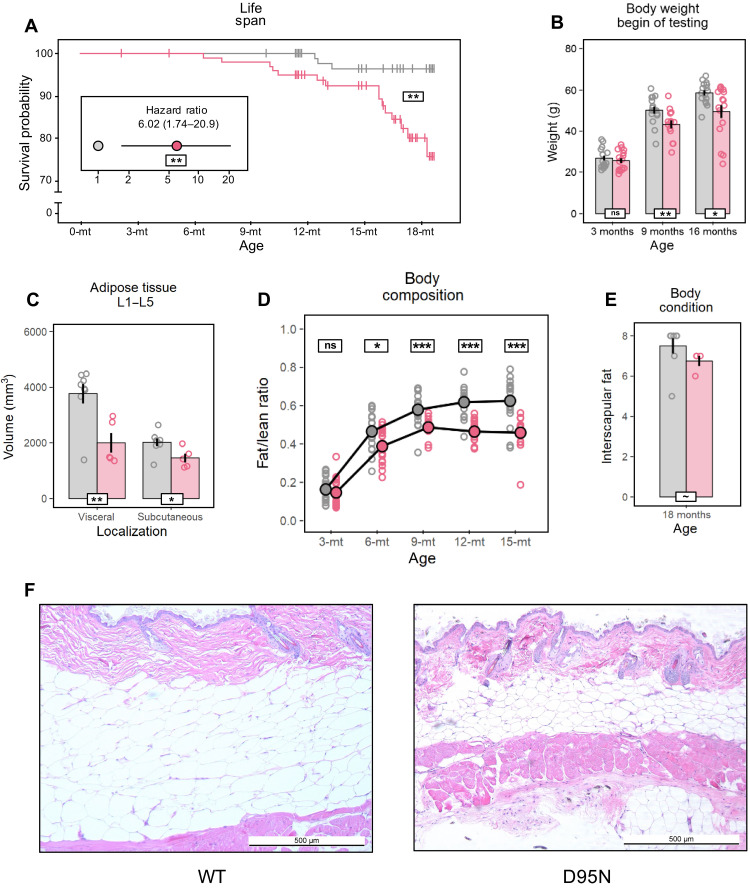
Reduced life span and altered body composition in *Rps9* D95N mice. Graphs in (B) to (D) and (F) show untransformed mean, SE, and individual data points. Red, *Rps9* D95N mutants; gray, WT. Genotype effect overall or split post hoc by age (white boxes) or age effect split post hoc by genotype (colored boxes): ***P < 0.001, **P < 0.01, *P < 0.05, ~*P* < 0.1, ns *P* ≥ 0.1. (**A**) Effect of the *Rps9* D95N mutation on survival (Kaplan-Meier: *P* = 0.0017) and hazard ratio of *Rps9* D95N versus WT mice (inset, Cox proportional hazards model: *P* = 0.005). Number of animals: WT, 66 F/60 M; *Rps9* D95N, 59 F/60 M. (**B**) Body weight (genotype: *F*_1,86_ = 14.09, *P* = 0.0003, η^2^ = 0.14; age: *F*_1,86_ = 214.9, *P* < 0.0001, η^2^ = 0.71; genotype × age: *F*_1,86_ = 4.348, *P* = 0.0400, η^2^ = 0.05). Number of animals: 3-month WT, 8 F/8 M; *Rps9* D95N, 8 F/8 M; 9-month WT, 8 F/8 M; *Rps9* D95N, 6 F/8 M; 16-month WT, 8 F/8 M; *Rps9* D95N, 8 F/8 M. (**C**) Adipose tissue distribution in 18-month male mice determined at levels L1 to L5 by micro-CT imaging (genotype visceral: *F*_1,11_ = 11.20, *P* = 0.0065; subcutaneous: *F*_1,11_ = 6.756, *P* = 0.0247). Number of animals: WT, 8 M; *Rps9* D95N, 5 M. (**D**) Repeated estimates of fat/lean mass ratio in male mice (age: *F*_1,508_ = 913.3, *P* < 0.0001, η^2^ = 0.49; genotype: *F*_1,33_ = 17.09, *P* = 0.0002, η^2^ = 0.32; genotype × age: *F*_1,138_ = 4.300, *P* = 0.0400, η^2^ = 0.02; Box-Cox λ-1.00 @ 0.03500). Number of animals: WT, 18 M; *Rps9* D95N, 18 M. (**E**) Interscapular fat score in male mice at sacrifice (genotype: *F*_1,10_ = 3.586, *P* = 0.0875, η^2^ = 0.18; Box-Cox λ 2.50 @ 4.0000). Number of animals: WT, 8 M; *Rps9* D95N, 4 M. (**F**) Representative hematoxylin and eosin–stained cross section of dorsal skin (interscapular area, ×50 magnification) from 18-month male WT (left) and *Rps9* D95N mice (right).

The life span of *Rps9* D95N mutant mice was significantly reduced according to Kaplan-Meier analysis, and their risk of dying prematurely was overall six times higher than in control animals (Fig. 3A). While only 3.5% of control mice were lost prematurely, all older than 12 months, 24% of the *Rps9* D95N mutant mice died prematurely. Their population started to decline already at 6.5 months with a marked acceleration at 16 months of age (Fig. 3A). Compared to control littermates, weight gain of the *Rps9* D95N mutant mice started to decelerate from 6 to 9 months of age (Fig. 3B). We performed quantitative assessments of body composition by Echo-MRI (magnetic resonance imaging) using male mice. Compared to control littermates and consistent with the overall reduction of body weight, we found decreased amounts of total body fat in the *Rps9* D95N mutants, together with a significantly reduced ratio of fat content to lean body mass in *Rps9* D95N animals starting from 6 to 9 months of age (Fig. 3D and fig. S5A). Analysis of fat distribution by micro–computed tomography (CT) imaging showed smaller amounts of subcutaneous and visceral fat in 18-month *Rps9* D95N mice (Fig. 3C), reflected also in reduced interscapular fat content at time of necropsy (18 months) (Fig. 3, E and F). A decrease in body weight and fat content is well documented in aging mice older than 1.5 years (*32*) and also occurs in humans older than 65 years (*33*, *34*).

**Fig. 4.**
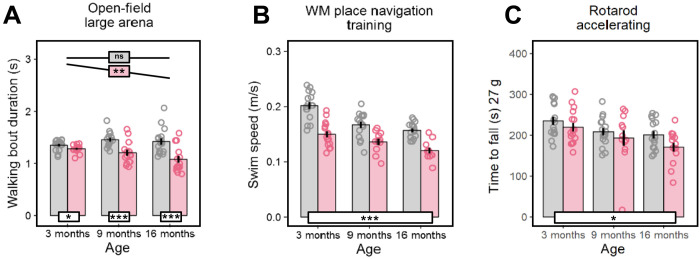
Deficient motor function in *Rps9* D95N mice. Graphs show untransformed mean, SE, and individual data points. Red, *Rps9* D95N mutants; gray, WT. Genotype effect overall or split post hoc by age (white boxes) or age effect split post hoc by genotype (colored boxes): ***P < 0.001, *P < 0.05, ~*P* < 0.1, ns *P* ≥ 0.1. (**A**) Average duration of walking bouts in the large open-field arena (genotype: *F*_1,83_ = 35.12, *P* < 0.0001, η^2^ = 0.30; age: *F*_1,83_ = 4.191, *P* = 0.0438, η^2^ = 0.05; genotype × age: *F*_1,83_ = 10.77, *P* = 0.0015, η^2^ = 0.11; Box-Cox λ = −0.50). (**B**) Average swim speed during testing in a water-maze (WM) task (genotype: *F*_1,78_ = 72.33, *P* < 0.0001, η^2^ = 0.48; age: *F*_1,78_ = 48.81, *P* < 0.0001, η^2^ = 0.38; genotype × age: *F*_1,78_ = 1.805, ns). (**C**) Time to fall off the accelerating rotarod, averaged over five trials and normalized by regression to a body weight of 27 g (genotype: *F*_1,82_ = 4.453, *P* = 0.0379, η^2^ = 0.05; age: *F*_1,82_ = 14.54, *P* = 0.0003, η^2^ = 0.15; genotype × age: *F*_1,82_ = 0.489, ns). Number of animals (A to C): 3-month WT, 8 F/8 M; *Rps9* D95N, 8 F/8 M; 9-month WT, 8 F/8 M; *Rps9* D95N, 6 F/8 M; 16-month WT, 8 F/8 M; *Rps9* D95N, 8 F/8 M.

Aging in rodents and humans is frequently accompanied by a loss in muscle function with decrements in muscle strength preceding muscle loss (*35*). In the absence of a significant reduction in lean mass (fig. S5B), the *Rps9* D95N mutants showed a slight impairment in tests related to motor function. Their walking bouts in a large open-field arena were significantly shortened (Fig. 4A), especially at older age. Furthermore, we observed a marked reduction of their swim speed while testing *Rps9* D95N mutant mice in a water-maze task (Fig. 4B). We also tested *Rps9* D95N mutant mice on the accelerating rotarod but unexpectedly found no significant impairment. Because performance on the rotarod is negatively correlated with body weight, we suspected that the reduced body weight of *Rps9* D95N mutant mice may have given them an advantage and masked a performance deficit. We used linear regression to normalize performance scores to a body weight of 27 g. The normalized data showed the expected decline of performance with age and revealed a mild deficit of *Rps9* D95N mutant mice (Fig. 4C). Together, these findings testify to a mild reduction of muscle strength in *Rps9* D95N mutants.

### *Rps9* D95N mice show hematopoietic alterations and enhanced levels of ROS and ROS-inflicted damage

**Fig. 5.**
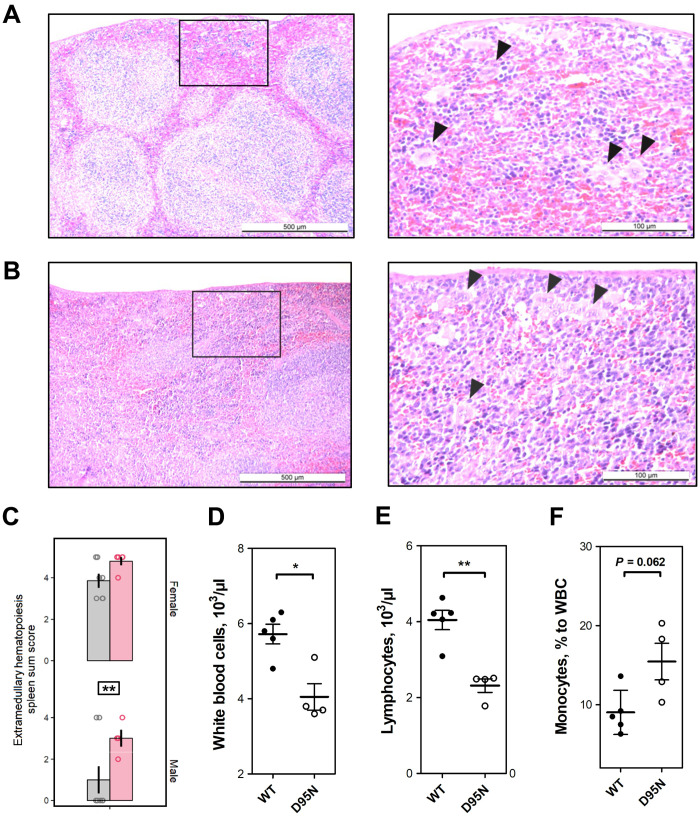
Hematopoietic alterations in *Rps9* D95N mutant mice. (**A** and **B**) Representative hematoxylin and eosin–stained section from the spleen of *Rps9* D95N mutant mice at 18 months of age; increased extramedullary hematopoiesis (EMH) is present in *Rps9* D95N mice (left: magnification ×50; right: magnification ×200). (A) Male *Rps9* D95N animal (score sum 3); (B) female *Rps9* D95N animal (score sum 5). EMH consists of groups of hematopoietic and myelopoietic cells spread within an area (quadrant in ×50 magnification); black arrows (in ×200 magnification) point toward megakaryocytes as the morphologically distinguishable characteristic of hematopoietic activity. (**C**) Extramedullary hematopoiesis in *Rps9* D95N mice at 18 months of age. Red, *Rps9* D95N; gray, WT. Untransformed mean, SE, and individual data points of sum score reflecting grade and distribution of EMH in the spleen (genotype: *F*_1,20_ = 8.734, *P* = 0.0078, η^2^ = 0.24; genotype × sex: *F*_1,21_ = 0.146, ns; Box-Cox λ 2.00 @ 0.00000). Genotype: **P < 0.01. Number of animals: WT, 7 F/8 M; *Rps9* D95N, 5 F/4 M. (**D** to **F**) Blood count analysis in female WT and *Rps9* D95N mice. (D) Number of white blood cells per microliter. (E) Number of lymphocytes per microliter. (F) Monocytes relative to white blood cells (WBC) (%). *P < 0.05 and **P < 0.01.

We next studied the hematopoietic system in mice at 18 months of age. Compared to control animals, the *Rps9* D95N mice showed evidence of increased extramedullary hematopoiesis in the spleen (Fig. 5, A to C). Blood count analysis was performed in female *Rps9* D95N mutants and revealed mild anemia together with significantly decreased white blood cell counts, lymphopenia, and relative monocytosis in comparison with WT female littermates at 18 months of age (Fig. 5, D to F, and table S1). Increased extramedullary hematopoiesis in the spleen is a common finding in aging mice (*32*), and anemia of unknown etiology is a frequent clinical problem in elderly humans (*36*). In addition, changes in the white blood system in female mice maintained until near end-of-life typically include lowered white blood cell counts, lymphopenia, and relative monocytosis (*30*). Together, these findings indicate that the hematopoietic system of 18-month *Rps9* D95N mutant mice shows changes characteristic of mice that are near the end-of-life.

**Fig. 6.**
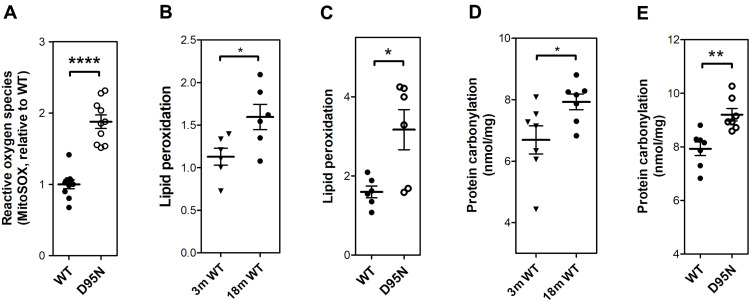
Increased levels of ROS and ROS-inflicted damage in *Rps9* D95N mutant mice. (**A**) Levels of mitochondrial ROS (superoxide anion radicals, MitoSOX assay) in skeletal muscle from mutant *Rps9* D95N mice in comparison to WT mice (*N* = 10; ± SEM). (**B**) Lipid peroxidation levels measured by colorimetric assay in skeletal muscle from 3-month-old WT compared to 18-month-old WT mice (*N* = 6; ± SEM). (**C**) Lipid peroxidation levels measured by colorimetric assay in skeletal muscle from 18-month-old *Rps9* D95N in comparison to age-matched WT mice (*N* = 6; ± SEM). (**D**) Protein carbonyl levels measured by enzyme-linked immunosorbent assay (ELISA) in heart tissue from 3-month-old WT compared to 18-month-old WT mice (*N* = 7; ± SEM). (**E**) Protein carbonyl levels measured by ELISA assay in heart tissue from 18-month-old WT and 18-month-old *Rps9* D95N mice (*N* = 7; ± SEM). *P < 0.05, **P < 0.01, and ****P < 0.0001.

A fraction of the oxygen consumed by cells results in production of superoxide in mitochondria, where it is dismutated to hydrogen peroxide by superoxide dismutase. The free radical theory of aging posits that aging is a result of progressive accumulation of reactive oxygen species (ROS)–mediated damage (*37*–*39*). We measured ROS produced by mitochondria isolated from muscle of mice aged 12 months and found that compared to WT animals, *Rps9* D95N mice showed significantly increased ROS levels (Fig. 6A). We further examined indirect markers of ROS-induced damage in female mice, first by measuring lipid peroxidation in skeletal muscle. In an age-dependent manner, lipid peroxidation increased in muscle of WT mice (Fig. 6B). Compared to WT littermates of identical age, muscle of 18-month *Rps9* D95N mutants showed significantly elevated levels of lipid peroxidation (Fig. 6C). We next assayed protein carbonylation, a marker of oxidative damage to proteins, in heart muscle. This follows observations in *Escherichia coli* that proteins synthesized by less accurate ribosomes show increased intrinsic sensitivity to oxidative damage (*40*, *41*). In an age-dependent manner, the amount of protein carbonyls increased in WT mice (Fig. 6D). Compared to age-matched WT mice, heart protein carbonyl levels in mutant *Rps9* D95N 18-month animals were significantly increased (Fig. 6E). Collectively, these findings point to enhanced levels of ROS and ROS-induced damage in the *Rps9* D95N mutants.

### *Rps9* D95N mice show epigenetic changes indicative of premature aging

**Fig. 7.**
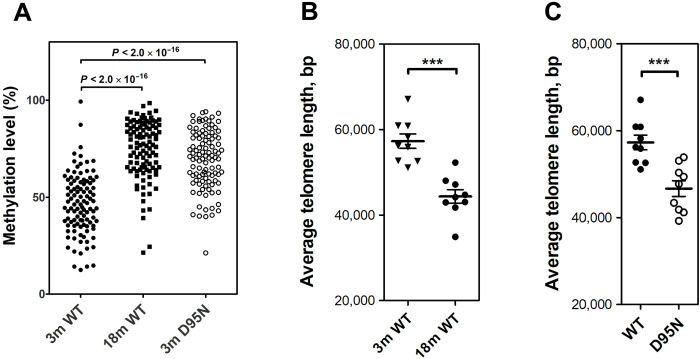
Epigenetic changes indicative of premature aging in *Rps9* D95N mutant mice. (**A**) Methylation levels (in %) of 104 age-related CpG sites in 3-month-old WT, 18-month-old WT, and 3-month-old *Rps9* D95N mutants (*N* = 4 for each group, each point represents mean; for further details, see fig. S6). For statistical calculations, the Kruskal-Wallis one-way analysis of variance was used. (**B**) Length of telomeres in brain of 3-month-old WT compared to 18-month-old WT mice (*N* = 9; ± SEM). (**C**) Length of telomeres in brain of 3-month-old WT and *Rps9* D95N mice (*N* = 9; ± SEM). ***P < 0.001.

Last, we wished to study premature aging in female mice using phenotype-independent epigenetic biomarkers. Telomere length and DNA methylation have been previously suggested to change with age (*42*–*44*). In humans, numerous CpG sites show DNA methylation patterns that change with age and retrospective analysis of longitudinal cohort studies have indicated that premature changes in DNA methylation–correlated molecular aging patterns coincide with a significantly increased risk of overall mortality (*45*). More recently, genome-wide age-related changes in DNA methylation have been studied in mouse liver testifying to epigenetic aging signatures and their slowdown by life span–extending interventions (*46*). We analyzed CpG methylation sites in liver DNA from female mice using whole-genome bisulfite sequencing (WGBS). We first established an age-related methylation signature within our cohorts of littermate control animals by assessing the liver methylome in WT animals at 3 and 18 months of age. This identified 760 CpG sites with age-associated differences in methylation. To study *Rps9* D95N–associated premature aging, we determined the methylation signatures in 3-month *Rps9* D95N mutant liver samples. Comparison to liver samples from 3-month WT animals identified 313 CpG sites with mutation-associated differences in methylation. Cross-comparison to the 760 age-associated CpG sites revealed that 104 of 313 mutation-related CpG sites (33%) corresponded to age-related CpG sites (*P* = 5.26 × 10^−46^ by hypergeometric test). Most notably, all of the 104 mutation-specific CpG sites that overlapped with the age-related CpG sites showed methylation changes concordant with premature epigenetic aging, i.e., sites that gained methylation in the mutants showed increased methylation upon aging, and sites that lost methylation in the mutants showed decreased methylation upon aging (see Fig. 7A and fig. S6). These data indicate that the 3-month *Rps9* mutant animals are significantly older in epigenetic age than their littermate age-matched controls.

In addition to changes in CpG methylation, replication-based telomere shortening is regarded as an epigenetic clock that triggers cellular senescence. We were particularly interested in assessing telomere instability in the brain, as in addition to mitosis-competent glial cells telomere instability in brain neuronal cells is intrinsic to brain aging beyond cell replication (*47*, *48*). We measured the average telomere length per chromosome end in the brain of female WT animals at 3 and 18 months of age using an established PCR-based assay. In an age-dependent manner, telomere length decreased in WT animals (Fig. 7B). We next assessed telomere length in the brain of 3-month female D95N animals. Compared to the age-matched WT animals, we observed a reduction in telomere length in the brain of 3-month *Rps9* D95N mutants (Fig. 7C).

## DISCUSSION

In general, the clustering of diseases, known as comorbidities, indicates a common factor. The comorbidities associated with aging, such as frailty, kyphosis, cataract, and skin dystrophia, imply a shared underlying mechanism. Thus, a holistic pathomechanistic view of aging and age-related diseases has to address systems-based rather than tissue- or organ-specific means of explanation. The idea that aging and the aging process may be related to protein synthesis and its accuracy has been an attractive hypothesis for many years, in part because the main contributor to errors in gene expression comes from mRNA translation by the ribosome with an average error rate of 10^−4^ (*1*, *2*). Starting with the early, but refuted error catastrophe hypothesis of aging back in the 1960s (*6*–*9*) to more recent purely descriptive findings that postulate a correlation of translational accuracy and maximum possible life span of an organism (*3*, *4*), this question has remained essentially unanswered, mainly due to the lack of an experimental model. We here filled this long-standing gap. Following identification of *Rps9* D95N as a ribosomal ambiguity mutation that confers error-prone protein synthesis in mammalian ribosomes, we successfully generated heterozygous *Rps9* D95N knock-in mice. Of interest, RPS9 D95N–mediated lifelong mistranslation mainly manifests at later age, as revealed by accelerated development of aging phenotypes and reduced life span in mice with RPS9 D95N–mediated organism-wide error-prone protein synthesis.

The genetic strategy we have chosen to manipulate translation contrasts with classical pathogenic concepts of ribosomal mistranslation that act at the level of individual aa-tRNA synthetases and that are detrimental for translation (*49*, *50*). Rather, we decided to increase the natural error rate in translation in a general, random, and stochastic manner by means of the RPS9 D95N ribosomal ambiguity mutation (*ram*). *Rams* affect the initial phase of tRNA selection, resulting in reduced discrimination against near-cognate aa-tRNAs, thus mimicking the mechanism of the ribosome’s inherent errors in protein synthesis. Structural analyses at atomic resolution will be required to pinpoint the molecular and mechanistic details of RPS9 D95N–mediated mistranslation.

In addition to reduced life span, characteristics of accelerated aging in the *Rps9* D95N mutant mice were present at multiple levels, i.e., morphological (altered fur, cataract, and hunchback posture), physiological (body composition and function, weight, fat mass, and muscle strength), and pathological (decreased life span, mouse urinary syndrome, and extramedullary hematopoiesis). With increased ROS production, heightened oxidative damage, telomere attrition, and altered DNA methylation, we identified biochemical changes that can act as cellular drivers of the accelerated aging observed in *Rps9* D95N mice. As a potential caveat to our interpretation, we note that we cannot formally rule out possible extraribosomal effects of the *Rps9* D95N allele in our transgenic mice. However, we consider this an unlikely possibility not the least because we have shown in our cell culture experiments that the mutant RPS9 protein readily becomes incorporated into ribosomes. In addition, increased error-prone translation was demonstrated both in *Rps9* D95N–transfected cell lines and in liver ribosomes from mutant D95N animals. Besides our results providing direct, experimental support for a link between translational accuracy, life span, and phenotypes present in aging animals, we also note that with this now available genetic model of ribosomal misreading, a possible connection between mistranslation and tumorigenesis, as suggested by findings of increased mutagenesis in mistranslating *E. coli* (*51*–*53*), becomes experimentally tractable.

## MATERIALS AND METHODS

### Vector construction and generation of transfected cell lines

Expression vectors pRps9-WT and pRps9-D95N were constructed by genOway, France. These vectors contain the mouse *Rps9* coding sequence (mouse and human RPS9 share an identical amino acid sequence) under control of the CAGGS promoter [chicken β-actin promoter coupled with cytomegalovirus (CMV) early enhancer] and the human growth hormone (hGH) polyadenylation signal. In brief, the mouse *Rps9* coding sequences [WT/D95N (GAT/AAC)] were amplified by PCR, and Sac I and Cla I restriction sites were introduced at the 5′ and 3′ ends, respectively. Using the Sac I and Cla I restriction sites, the 627-nucleotide-sized fragments were each ligated into the expression vector downstream of the constitutive CAGGS promoter and transcriptionally linked to IRES–enhanced GFP for visualization of target gene expression. In addition, a HygR cassette from plasmid pGL4.14 (Promega) was inserted at the Mlu I restriction site. Successful ligation resulted in plasmids pRps9-WT-IRES-GFP-HygR and pRps9-D95N-IRES-GFP-HygR, respectively.

HEK293 cells were transfected with pRps9(WT/D95N)-IRES-GFP-HygR expression vectors using Xfect (Takara) and cultured in Dulbecco’s modified Eagle’s medium (DMEM) supplemented with 10% fetal bovine serum (FBS) under hygromycin B selection (100 μg/ml) for 5 to 7 weeks. GFP-expressing colonies were picked for further characterization. GFP fluorescence was analyzed by flow cytometry using BD FACSCanto II (BD Biosciences) and the FlowJo data analysis software (Tree Star Inc., Ashland, OR). Transgene expression levels were confirmed using TaqMan RT-qPCR.

### Determination of *Rps9* mRNA expression in transfected cell lines

mRNA was isolated using TRIzol (Thermo Fisher Scientific) according to the manufacturer’s protocol. qRT-PCR was used to determine the relative ratio of *Rps9* transgene to endogenous mRNA using species-specific *Rps9* primers flanking the site of mutation. Transgenic (mouse) and endogenous (human) *Rps9* share identical amino acid sequences, but their nucleotide sequences are different. Discrimination between WT and D95N mutant *Rps9* transgene was achieved using TaqMan probes specific to WT (5′-GATGAAGCTGGATTAC-3′, NED-labeled) or mutant (5′-CAAGATGAAGCTGAACT-3′ for D95N, FAM-labeled) sequences. Experiments were conducted in triplicates using the TaqMan Kit (Life Technologies) and the ABI 7500 Fast Real-Time PCR System (Life Technologies). Amplification was 40 cycles (95°C for 20 s and 60°C for 45 s); the ratio of transgene versus endogenous *Rps9* was calculated as previously described (*54*).

### Dual-luciferase mistranslation reporter assay

Mistranslation was assessed as described previously (*55*, *56*). In brief, misreading was determined using the pRM hRluc-hFluc H245R vector, where His^245^ (CAC codon) was replaced by Arg^245^ (near-cognate CGC codon or noncognate AGA codon). Read-through was determined using pRM hRluc-hFluc D357X, where Asp^357^ (GAC codon) was replaced by a UGA nonsense codon in the firefly luciferase (Fluc) transcript. Cells were transfected using Xfect (Takara) according to the manufacturer’s protocol. After 24-hour incubation, cells were lysed and luminescence was measured using the FLx800 luminometer (Bio-Tek Instruments). Renilla luciferase (Rluc) activity was used as an internal control. Relative misreading and read-through were calculated by the ratio of D95N mutant Fluc/Rluc activity to WT Fluc/Rluc activity. For geneticin treatment, cells were transfected, incubated overnight, and then treated with the drug for further 24 hours before lysis and measurement of luciferase activity.

### In vivo ^35^S-Met incorporation

HEK293 cells were grown in 24-well plates in DMEM with 10% FBS to 80% confluence, washed with phosphate-buffered saline (PBS), and incubated for 30 min in methionine-free RPMI. The medium was replaced with methionine-free RPMI supplemented with 10 mCi of ^35^S-methionine (Hartmann) and incubated at 37°C for the indicated time periods. Translation was stopped by the addition of a stop medium [DMEM supplemented with cycloheximide (100 μg/ml) and cold methionine (1 mg/ml)]. Cells were detached by pipetting and washed once with 1× PBS before lysing with 1× Passive Lysis Buffer (Promega). Proteins were precipitated with 10% trichloroacetic acid (Sigma-Aldrich), and precipitates were collected on GF/C filter membranes (PerkinElmer). The ^35^S-Met incorporation was measured by scintillation counting (PerkinElmer Trilux MicroBeta 96-well format counter).

### Polysome extraction, profiling, and polysome run-off assays

Polysome extractions were performed according to (*57*) with modifications. A petri dish with HEK293 cells (~80% confluence) was preincubated for 10 min at 37°C with cycloheximide (100 μg/ml). All subsequent steps were performed on ice. Cells were collected in 1.5-ml tubes and washed with ice-cold PBS supplemented with cycloheximide (100 μg/ml). Cells were pelleted at low speed (500*g* for 5 min), resuspended in lysis buffer [20 mM Hepes (pH 7.5), 10 mM KCl, 3 mM Mg-Ac, and 1 mM dithiothreitol (DTT)] at a 1:3 ratio, and lysed by passing the suspension rapidly through a 0.45-mm needle 15 times. After lysis, 10× Polysome Extraction Buffer [0.2 M Hepes (pH 7.5), 40 mM Mg-Ac, 1.2 M K-Ac, and 10 mM DTT] was added immediately to the lysate to a final concentration of 1×. The lysate was centrifuged at 1000*g* for 10 min. The supernatants were moved to fresh tubes and centrifuged further at 16,000*g* for 10 min. The final supernatants containing the polysomes were recovered. One OD_260_ (optical density at 260 nm) unit was loaded onto a sucrose gradient column (8 to 50%), ultracentrifuged (SW41 rotor, 39,000 rpm, 4°C) for 3 hours, and analyzed using a Teledyne ISCO Polysome Profiler System. Polysome run-off assays were used to determine polysomal half-life as described previously (*24*).

### Aggregation assays

For luciferase-based aggregation assay, stable *Rps9* transgenic cell lines were transiently transfected with a eukaryotic expression vector carrying the firefly luciferase reporter under the control of cytomegalovirus promotor (pGL4.50, Promega). Cells were suspended in lysis buffer [20 mM Hepes (pH 7.5), 10 mM KCl, 3 mM Mg-Ac, and 1 mM DTT] at a 1:3 ratio, lysed by passing the suspension rapidly through a 0.45-mm needle 15 times, and fractionated by centrifugation (16,000*g* for 25 min) into soluble and pellet fractions. The resulting fractions were analyzed by Western blot using horseradish peroxidase (HRP)–conjugated specific anti-Fluc antibodies (Abcam). The signal intensity of the luciferase bands in the different fractions was quantified using ImageQuant (GE Healthcare), and the ratio between pellet and soluble fractions was calculated.

Aggregation of endogenous intracellular proteins was assessed using a PROTEOSTAT aggresome detection kit (Enzo Life Sciences) according to the manufacturer’s protocol. Treatment with an MG-132 proteasome inhibitor (10 μM, 15 hours) was used as a positive control. Fixed (3% paraformaldehyde) and stained cells were analyzed by flow cytometry using fluorescence-activated cell sorting (FACS) LSRFortessa (BD Biosciences) for measurements and FlowJo software for analysis of the FACS data.

### Generation of knock-in *Rps9* transgenic line

The *Rps9* D95N mutant mice were generated by genOway, France, using a knock-in targeting vector displaying a mutated exon 4 (D95N; GAT-AAC) and a neomycin selection cassette flanked by *lox*P sites (fig. S4). The gene-targeting vector was constructed from genomic C57BL/6 mouse strain DNA. Linearized targeting vector was transfected into C57BL/6 ES cells, and positive selection by addition of G418 was started 48 hours after electroporation. Resistant clones were isolated, amplified, duplicated, and genotyped by both PCR and Southern blot analysis.

*Rps9*-targeted cells were characterized using the following primers: 175557sa-EBO11: 5′-GGACAGCAAATCAACAGTCCTAGAAGCTC-3′ and 175556sa-EBO11: 5′-ATCCGTAATCGACTTCAAGGTGCTCC-3′. The corresponding PCR amplicon of 2953 bp was sequenced to detect the targeted insertion of the D95N mutation at the *Rps9* locus. Gene targeting was confirmed by Southern blot analysis using internal and external probes on both 5′ and 3′ ends. PCR and Southern blot genotyping led to the identification of five targeted clones. Recombined ES cell clones were microinjected into C57BL/6 blastocysts and gave rise to male chimeras. Breeding with C57BL/6 CRE deleter mice (CMV-Cre) produced the heterozygous *Rps9* D95N mutant mice devoid of selection cassette. These heterozygous *Rps9* mutants (*Rps9^D95N/WT^*) are referred to as *Rps9* D95N mutant mice (fig. S4). Heterozygous mice were genotyped by PCR, Southern blot, and sequencing.

*Rps9^D95N/WT^* mice were first identified by PCR using primer 175549cof-EBO11: 5′-CCTGATAACTTTTCTTCCCAGAGCCCTA-3′ and primer 175548cof-EBO11: 5′-GTAGTCTGAGGAATAAATGTGTGCTGTTTGTG-3′. These primers result in amplification of a specific 269-bp fragment for the *Rps9^WT^* allele and a specific 324-bp fragment for the *Rps9^D95N^* allele.

PCR-positive heterozygous *Rps9^D95N/WT^* mice were confirmed by Southern blot analysis using the 3′ external probe (amplified using the following primers: 5′-AGTTAGCACTCAATGTAAAACCTTCCCTTCC-3′/5′-CTGAACACAGAATATGAACAAGCAGAAGAGG-3′). Upon digestion with PflF1, 8.2- and 7.6-kb fragments are expected for *Rps9^WT^* and *Rps9^D95N^* alleles, respectively (fig. S4).

The presence of the mutation in *Rps9^D95N/WT^* mice was confirmed by RT-PCR and sequencing (fig. S4). Mice tissue sections were frozen until RNA extraction. RNA was extracted using TRIzol reagent (Thermo Fisher Scientific) according to the manufacturer’s instructions. The purified RNA was deoxyribonuclease (DNase)–treated, and complementary DNA (cDNA) synthesis was performed using a SuperScript II cDNA synthesis kit (Life technologies) using random hexamers and 40 ng of RNA in 20 μl of reagent according to the manufacturer’s instructions. The following primer pairs were used for RT-PCR and sequencing: EBO11_ex1: 5′-CAGAAGCTGGGTTTGTCGCA-3′ and EBO11_ex5: 5′-TCCTCTTCCTCATCATCACCAGC-3′. These primers result in amplification of a specific 570-bp fragment.

Cohorts of animals were produced by standard breeding or in vitro fertilization (IVF). For IVF, females’ superovulation was induced by administration of CARD HyperOva (Cosmo Bio. Ltd.) to 26- to 30-day-old females (0.1 or 0.2 ml). Forty-eight hours later, 7.5 IU of human chorionic gonadotropin (hCG; Sigma-Aldrich) was intraperitoneally injected to these mice. Superovulated females were sacrificed approximately 17 hours after administrating hCG, and their oviducts were quickly collected and transferred to a fertilization dish covered with paraffin oil. Under microscopic observation, cumulus-oocyte complexes were collected from the oviducts and transferred to a 200-μl drop of fertilization medium. The number of ovulated oocytes in each group and their fertilization ability were examined. For collection of spermatozoa, males’ cauda epididymides were recovered and transferred to a dish of sperm preincubation medium (FERTIUP, Cosmo Bio. Ltd.) covered with paraffin oil. Clots of sperm were collected from the cauda epididymides using a dissecting needle and transferred to a 100-μl drop of sperm preincubation medium for 60 min. Sperm suspension was then added to the fertilization drop containing cumulus-oocyte complexes and cultured with oocytes for 3 hours at 37°C. Then, oocytes were washed in three drops of human tubal fluid (80 μl). For insemination, freshly harvested two-cell embryos were transferred into the oviducts of pseudo-pregnant females.

### Extraction of ribosomes from liver tissue and in vitro stop codon read-through translation assay

mRNAs for firefly D357X_TGA_ and renilla reporters were prepared by in vitro transcription using T7 RNA polymerase as described in (*55*). To increase translation efficiency of the Fluc D357X_TGA_ mRNA template, we inserted the translational enhancer sequence from tobacco mosaic virus into the 5′ untranslated region of the gene (*58*). Extraction of active ribosomes from liver and in vitro translation assays were performed as described in (*59*) with some modifications. In brief, deep-frozen mouse liver was grinded under liquid nitrogen. The tissue (0.5 g) was transferred to 1 ml of buffer R2 [20 mM Hepes (pH 7.5), 10 mM NaCl, 25 mM KCl, 1.1 mM MgCl_2_, and 7 mM 2-mercaptoethanol] supplemented with cOmplete protease inhibitor cocktail (Roche) and carefully homogenized using a 2-ml glass homogenizer. Debris and fat were removed by two consecutive low-speed centrifugations (5000*g* for 5 min, 12,000*g* for 10 min), and the ribosomes were collected by high-speed centrifugation (200,000*g* for 90 min). The ribosomal pellet was rinsed with buffer R [10 mM Hepes (pH 7.5), 10 mM CH_3_CO_2_K, 1 mM (CH_3_CO_2_)_2_Mg, 1 mM DTT, and cOmplete protease inhibitor cocktail], dissolved in 150 μl of the same buffer, and stored at −80°C. Concentration of ribosomes was determined by absorbance *A*_260_ (1 *A*_260_ unit corresponds to ~20 pmol of 80*S* ribosomes). Ribosome-free cell lysate (S100_RRL_) was prepared as follows: 1 ml of nuclease-treated commercial RRL (Promega) was supplemented with 10 μM Hemin (Sigma-Aldrich), 25 μg of creatine phosphokinase (Sigma-Aldrich), 5 mg of creatine phosphate (Sigma-Aldrich), 50 μg of bovine liver tRNAs (Sigma-Aldrich), and 3 mM d-glucose (Sigma-Aldrich). After centrifugation for 2 hours and 30 min at 240,000*g*, postribosomal supernatant was collected, frozen, and stored at −80°C. In vitro translation mixture contained the following components: 15 μl of S100_RRL_, amino acids (100 μM each; Sigma-Aldrich), 1 μl of RiboLock (Thermo Fisher Scientific), 2 μg of mRNA Fluc D357X_TGA_, 2 μg of mRNA Rluc, and 1 pmol of ribosomes in 30-μl final volume. Reaction was incubated for 45 to 50 min at 37°C, and activities of firefly and renilla luciferases were measured. Relative read-through was calculated by the ratio of Fluc/Rluc activity.

### Animal experiments

Animal housing and all experimental procedures have been in accordance with the Swiss Animal Protection Law and have been approved by the Cantonal Veterinary Office of Zurich, Switzerland. Mice were housed under a 12/12-hour light-dark cycle (lights on at 20:00) in groups of two to five, unless individual housing was required by experimental protocols. Mice were tested during the dark phase of the cycle under indirect dim light (ca. 12 lux) unless specified otherwise. Three cohorts comprising a total of 92 mice were examined in the same sequence of behavioral tests (in the order listed below) starting at the age of 3 (32 mice), 9 (32 mice), and 16 (32 mice) months and lasting approximately 2 months. Each cohort contained *Rps9*^D95N/WT^ mutant mice (D95N) and littermate *Rps9*^WT/WT^ controls in approximately balanced numbers of both sexes. The sequence of tests was as follows: large open field, water-maze place navigation, and rotarod. Two female D95N mice of the 9-month cohort were retrospectively excluded from all analyses because their genotype could not be verified.

### Rotarod

The digitally controlled Ugo Basile Mouse Rota-Rod apparatus (catalog no. 47600, Ugo Basile, 21025 Comerio VA, Italy; www.ugobasile.com) permitted to test five mice concurrently. The diameter of the drum was 30 mm. Six dividers with 25 cm diameter confined five lanes, each 57 mm wide. During each trial, the drum was accelerating from 4 to 40 rpm over the course of 5 min. The latency until a mouse fell off the drum was recorded using a trip switch below the animals. Each mouse was submitted to five trials with an intertrial interval of at least 30 min.

### Video tracking

During open-field and water-maze tests, the mice were video-tracked using a Noldus EthoVision XT 11.5 system (Noldus Information Technology, Wageningen, The Netherlands; www.noldus.com). The system recorded position, object area, and the status of defined event recorder keys on the keyboard. Raw data were then transferred to public domain software Wintrack 2.4 (www.dpwolfer.ch/wintrack) for further analysis.

### Large open field

The round arena had a diameter of 150 cm and 35-cm-high sidewalls made of white polypropylene (*60*). Each subject was released near the wall and observed for 10 min on two subsequent days. Vertical activity was estimated by counting reductions of object size in the absence of locomotion.

### Water-maze place navigation

The round white poly-propylene pool had a diameter of 150 cm with 68-cm-high walls. It was filled with water (24° to 26°C, depth 15 cm), which was rendered opaque by addition of 1 liter of milk (UHT whole milk 3.5% fat, Coop, Switzerland). The white quadratic goal platform (14 cm × 14 cm) was made of metallic wire mesh and painted white. It was hidden 0.5 cm below the water surface in the center of one of the four quadrants, approximately 30 cm from the side wall. Salient extra-maze cues were placed on the walls of the testing room. Animals performed 30 training trials (maximum duration, 120 s), 6 per day with intertrial intervals of 30 to 60 min and varying starting positions. During the first 18 trials, the hidden platform was held in the same position (acquisition phase) and then moved to the opposite quadrant for the remaining 12 trials (reversal phase). The first 60 s of the first reversal trial served as probe trial to test for spatial retention.

### Statistical analysis

Statistical analyses and graphs were produced using R version 4.0.5, complemented with the packages ggplot2, psych, moments, survival, and survminer. Unless specified otherwise, data were analyzed using a general linear model, with genotype (*Rps9* D95N, WT) and sex [female (F), male (M)] as between-subject factors. Age cohort (3, 9, and 16 months) was introduced as additional between-subject factor if multiple age cohorts had been analyzed. A within-subject factor for age was added instead if measurements had been repeated at multiple ages. Significant interactions were resolved by splitting the model. Significant effects of (within-subject) factors with >2 categorical levels were further explored using pairwise *t* tests. Variables with strongly skewed distributions or strong correlations between variances and group means were subjected to Box-Cox transformation before statistical analysis, as specified in figure legends. The statistical significance threshold was set at 0.05. The false discovery rate (FDR) control procedure of Hochberg was applied to groups of conceptually related variables within single tests to correct significance thresholds for multiple comparisons. Similarly, FDR correction was applied during post hoc testing.

### Body composition and tissue processing for histopathology

We performed trimonthly quantitative assessments of body composition in conscious male mice with a body composition analyzer (EchoMRI-500, EchoMRI LLC, Houston, TX, USA) starting at the age of 3 months. In addition, at 16 months of age, the distribution of body fat was analyzed using micro-CT imaging (Quantum Fx, PerkinElmer, Waltham, MS, USA). Animals were briefly anesthetized with gas anesthesia, whole-body scans were performed at 50 kV/160 mAs, and the body segment containing the lumbar vertebrae L1 to L5 was then analyzed for fat distribution using Analyze 12.0 (AnalyzeDirect, Overland Park, KS, USA).

Following necropsy at 18 months of age, dorsal skin (interscapular area) and spleen were fixed in 10% buffered formalin for paraffin embedding. Samples were trimmed, dehydrated through ascending alcohol rows, and subsequently embedded in paraffin. Histological sections were prepared (3 to 5 μm thickness) and stained with hematoxylin and eosin. A four-step semiquantitative grading system was used to categorize the histologic alterations at a quantitative level: (i) severity of changes observed (1 = minimal, 2 = mild, 3 = moderate, 4 = marked); (ii) distribution of changes within the tissue (1 = focal, 2 = multifocal, 3 = coalescing, 4 = diffuse). Combination of these two criteria gave the score sum.

### Blood count analysis

Blood count analysis was performed by the Veterinary Clinical Laboratory, University of Zurich. EDTA blood samples were analyzed within 6 hours after collection using the automated hematology analyzer Sysmex XT-2000iV. Data are given in table S1.

### Preparation of muscle homogenates and mitochondrial ROS measurements

Red muscle was removed from quadriceps and gastrocnemius (~10 to 100 mg) and washed in 5 ml of ice-cold PBS/10 mM EDTA. Visible fat, ligaments, and connective tissue were removed by trimming the muscle, which was then minced into small pieces. Cleaned muscle tissue was then transferred to an ice-cold solution of PBS/10 mM EDTA plus 0.05% accutase and incubated for 30 min (*61*). After centrifugation at 200*g* for 5 min at 4°C, the pellet was resuspended in 1 ml of Hanks’ balanced salt solution (HBSS) and homogenized with a glass homogenizer (10 to 15 strokes, 400 rpm). Protein content was normalized to 2 mg/ml protein in HBSS, and 200 μl of the solution was transferred to 1.5-ml black Eppendorf tubes (*62*). Mitochondrial ROS levels were assessed using the fluorescent dye Red Mitochondrial Superoxide Indicator (MitoSOX, final concentration 5 μM). After 45-min incubation at 37°C, muscle homogenate was centrifuged at 500*g* for 3 min and washed with 250 μl of HBSS. The centrifugation and washing steps were repeated twice. Last, the pellet was resuspended in 200 μl of HBSS, and 100 μl of the preparation was transferred to a black 96-well plate (in duplicates). MitoSOX triggers the formation of red fluorescent products that were detected at 531 nm (excitation)/595 nm (emission). The intensity of fluorescence is proportional to mitochondrial ROS levels. The fluorescence was measured using a Cytation 3 multimode plate reader (BioTek).

### Lipid peroxidation

Total levels of lipid peroxidation were measured in extracts of skeletal muscle (quadriceps) using a Lipid Peroxidation (MDA) Assay kit (Abcam) according to the manufacturer’s protocol. Muscle tissue (15 to 20 mg) was homogenized in lysis solution, and the protein concentration was determined by measuring OD_260_. The malondialdehyde (MDA) absorbance was measured on a microplate at 532 nm using a Cytation 5 multifunctional plate reader (BioTek). MDA absorbance measured was normalized to protein content to calculate absolute levels of lipid peroxidation.

### Measurement of protein carbonyls

Levels of protein carbonyls were assessed in heart protein extracts using the Protein Carbonyl ELISA Kit (Abcam) according to the manufacturer’s instructions. In brief, protein samples were adjusted to 10 μg/ml and allowed to adhere overnight at 4°C to wells of a 96-well plate provided by the manufacturer. Subsequently, reaction with dinitrophenylhydrazine (DNPH) was performed for derivatization of the protein carbonyl groups. The DNP-modified proteins were probed with anti-DNP antibody and developed with secondary HRP-conjugated antibody. Following substrate addition, absorbance (OD_450_) was measured with a Cytation 5 multifunctional plate reader (BioTek) and carbonyl content in protein samples was determined by comparing its absorbance with that of a known reduced/oxidized bovine serum albumin standard curve. Each sample was measured in triplicates.

### WGBS sequencing and data processing

Genomic DNA was sheared, end-repaired, and adapter-ligated using methylated adapters that are protected from bisulfite conversion. Subsequently, the adapter-ligated DNA was bisulfite-converted, which chemically converts all unmethylated cytosines into the RNA base uracil. In the subsequent PCR, all uracils were replaced by thymidine (the DNA analog of uracil). The libraries were then sequenced on an Illumina NovaSeq 6000 system at the Eurofins Genomics Sequencing Europe GmbH, Germany, generating 150-bp paired-end bisulfite-converted sequencing reads for each sample.

Sequencing reads were analyzed with DRAGEN Methylation pipeline (v3.7.5) using mouse genome version GRCm38/mm10 (http://edicogenome.com/dragen-bioit-platform/). DRAGEN Methylation pipeline is built on the principles of BISMARK to accurately align bisulfite-converted sequencing reads from WGBS to reference mouse genome using FPGA processors for acceleration. Briefly, sequencing reads were aligned to bisulfite-converted reference GRCm38 mouse genome, the alignments were calculated for both Watson and Crick strands, and the highest quality unique alignment was retained. A genome-wide cytosine methylation report was generated by DRAGEN to record counts of methylated and unmethylated cytosines at each cytosine position in the genome. Methylation counts for the CpG cytosine context were recorded. DNA methylation level of each CpG was calculated by the number of methylated reads over the total number of sequenced reads, and the CpG sites with less than 10× read coverage were not included in the downstream analysis. Filtering of data to identify sites that were reliably measured with sufficient sequencing depth and present in every replicate yielded 184.497 CpG sites for the age-related comparison (18-month versus 3-month animals, 4 animals each) and 149.803 CpG sites for the mutation-related comparison (3-month mutant versus 3-month WT, 4 animals each). A total of 142.782 CpG sites were shared between the age-related and the mutation-related comparisons. Differential methylation analysis was performed using methylation calls within a CpG context from all samples using R package methylKit v1.17.3 (*63*). Pair-wise differential CpG methylated sites were obtained applying Fisher’s exact test to compute *P* values, followed by adjustments for FDR using the SLIM method. Significant differential CpG methylation sites were identified by using 1% FDR and minimum of 25% methylation level as cutoff values. Further comparative analysis was done using custom scripts written in R v3.6.3. The methylation dataset is given in tables S2 to S4.

### Telomere length measurement

The length of telomeres was assessed using the Absolute Mouse Telomere Length Quantification qPCR Kit (ScienCell) according to the manufacturer’s protocol. Total genomic DNA was extracted from mouse brain using a DNeasy kit (Qiagen). Every PCR contained 2 ng of genomic DNA as a template. Every experimental reaction with extracted brain DNA was analyzed in duplicates. The reference mouse genomic DNA was analyzed in triplicates. Calculation of the telomere length was done according to (*64*).

### Ethical animal research statement

All experiments performed on *M. musculus* C57BL/6 complied with ethical regulations for animal testing and research and were approved by the Veterinary Office of the Canton of Zurich (licenses ZH29/2012, ZH41/2018, and ZH207/2019).

## Notes

View/request a protocol for this paper from *Bio-protocol*.
