# Supplementary material for: Premature aging in mice with error-prone protein synthesis
Source: Sci Adv. 2022 Mar 2;8(9):eabl9051. doi: 10.1126/sciadv.abl9051 (PMC8890705; doi:10.1126/sciadv.abl9051)
Supplement: Supplementary file 1 — Figs. S1 to S6 Table S1 [file sciadv.abl9051_sm.pdf]

Science Advances
AAAS

Supplementary Materials for

Premature aging in mice with error-prone protein synthesis

Dimitri Shcherbakov, Martina Nigri, Rashid Akbergenov, Margarita Brilkova, Matilde Mantovani, Patricia Isnard Petit, Amandine Grimm, Agnieszka A. Karol, Youjin Teo, Adrián Cortés Sanchón, Yadhu Kumar, Anne Eckert, Kader Thiam, Petra Seebeck, David P. Wolfer, Erik C. Böttger*

*Corresponding author. Email: boettger@imm.uzh.ch

Published 2 March 2022, *Sci. Adv.* **8**, eabl9051 (2022)
DOI: 10.1126/sciadv.abl9051

The PDF file includes:

Figs. S1 to S6
Table S1
Legends for tables S2 to S4

Other Supplementary Material for this manuscript includes the following:

Tables S2 to S4

Fig. S1.

```
         1                                      40                                      80
   MUS:  MPVARSWVCR KTYVTPRRPF EKSRLDQELK LIGEYGLRNK REVWRVK--F TLAKIRKAAR ELLTLDEKDP RRLFEGNALL  :78
   RAT:  MPVARSWVCR KTYVTPRRPF EKSRLDQELK LIGEYGLRNK REVWRVK--F TLAKIRKAAR ELLTLDEKDP RRLFEGNALL  :78
BOVINE:  MPVARSWVCR KTYVTPRRPF EKSRLDQELK LIGEYGLRNK REVWRVK--F TLAKIRKAAR ELLTLDEKDP RRLFEGNALL  :78
   MAN:  MPVARSWVCR KTYVTPRRPF EKSRLDQELK LIGEYGLRNK REVWRVK--F TLAKIRKAAR ELLTLDEKDP RRLFEGNALL  :78
 DANIO:  MPVAKTWVCS KTYVTPRRPF EKSRLDQELR LIGEYGLRNK REVWRVK--F TLAKIRKAAR ELLTLDEKDP KRLFEGNALL  :78
 YEAST:  MPRAP-RTYS KTYSTPKRPY ESSRLDAELK LAGEFGLKNK REIYRIS--F QLSKIRRAAR DLLTRDEKDP KRLFEGNALI  :77
   ECO:  ---------M ARYLGPKLKL SRREGTDLFL KSGVRAIDTK CKIEQAPGQH GARKPRLSDY GVQLREKQKV RRIYG---VL  :68

                          Yeast D94N
                          Mouse D95N
        81                ↓                    120                                     160
   MUS:  RRLVRIGVLD EGKMKLDYIL GLKIEDFLER RLQTQVFKLG LAKSIHHARV LIRQRHIRVR KQVVNIPSFI VRLD------  :152
   RAT:  RRLVRIGVLD EGKMKLDYIL GLKIEDFLER RLQTQVFKLG LAKSIHHARV LIRQRHIRVR KQVVNIPSFI VRLD------  :152
BOVINE:  RRLVRIGVLD EGKMKLDYIL GLKIEDFLER RLQTQVFKLG LAKSIHHARV LIRQRHIRVR KQVVNIPSFI VRLD------  :152
   MAN:  RRLVRIGVLD EGKMKLDYIL GLKIEDFLER RLQTQVFKLG LAKSIHHARV LIRQRHIRVR KQVVNIPSFI VRLD------  :152
 DANIO:  RRLVRIGVLD EGKMKLDYIL GLKVEDFLER RLQTQVFKLG LAKSIHHARV LIRQRHIRVR KQVVNIPSFV VRLD------  :152
 YEAST:  RRLVRVGVLS EDKKKLDYVL ALKVEDFLER RLQTQVYKLG LAKSVHHARV LITQRHIAVG KQIVNIPSFM VRLD------  :151
   ECO:  ERQFRN--YY KEAARLKGNT GENLLALLEG RLDNVVYRMG FGATRAEARQ LVSHKAIMVN GRVVNIASYQ VSPNDVVSIR  :146

       161                                     201
   MUS:  ----SQKHID FSLRSPYGGG RPGRVKRKN- -----AKKGQ GGAGAGDDEE ED--------  :194
   RAT:  ----SQKHID FSLRSPYGGG RPGRVKRKN- -----AKKGQ GGAGAGDDEE ED--------  :194
BOVINE:  ----SQKHID FSLRSPYGGG RPGRVKRKN- -----AKKGQ GGAGAGDDEE ED--------  :194
   MAN:  ----SQKHID FSLRSPYGGG RPGRVKRKN- -----AKKGQ GGAGAGDDEE ED--------  :194
 DANIO:  ----SQKHID FSLRSPYGGG RPGRVKRKN- -----AKKAQ GGGGGGDDEE ED--------  :194
 YEAST:  ----SEKHID FAPTSPFGGA RPGRVARRN- -----AARKA EASGEAAEEA EDEE------  :195
   ECO:  EKAKKQSRVK AALELAEQRE KPTWLEVDAG KMEGTFKRKP ERSDLSADIN EHLIVELYSK  :206
```

Fig. S1. Alignment of eukaryotic uS4 (RPS9) ribosomal proteins. Conserved amino acids are shown in red. Position of D95N mutation (D94N in yeast) is highlighted.

Fig. S2.

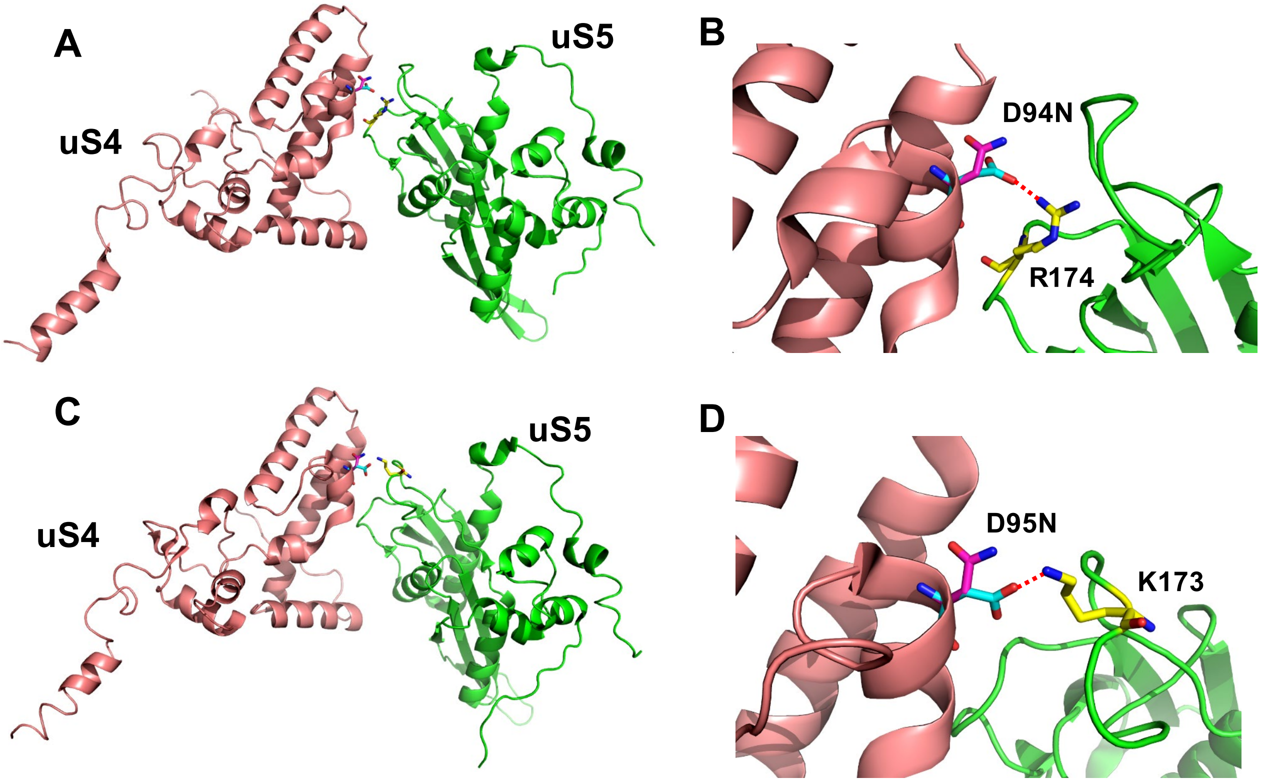

Fig. S2. Modelling uS4 (RPS9) D95N mutation at the uS4-uS5 (RPS9-RPS2) interface based on available crystal structures. uS4 (RPS9) and uS5 (RPS2) are shown in rose and green respectively for all diagrams. (**A**) Protein-protein interface uS4-uS5 of the yeast ribosome (Source – PDB 4V7R). Modelling of D94N substitution is shown in magenta. (**B**) Position and orientation of native uS4-D94 residue (cyan) making polar contact with uS5-R174 (yellow); D94N substitution is shown in magenta. (**C**) Protein-protein interface uS4-uS5 of the human ribosome (Source - PDB 4V6X). Modelling of D95N substitution is shown in magenta. (**D**) Position and orientation of native uS4-D95 residue (cyan) making polar contact with uS5-K173 (yellow). PyMol v1.8 (Schrödinger Inc.) was used for modelling.

Fig. S3.

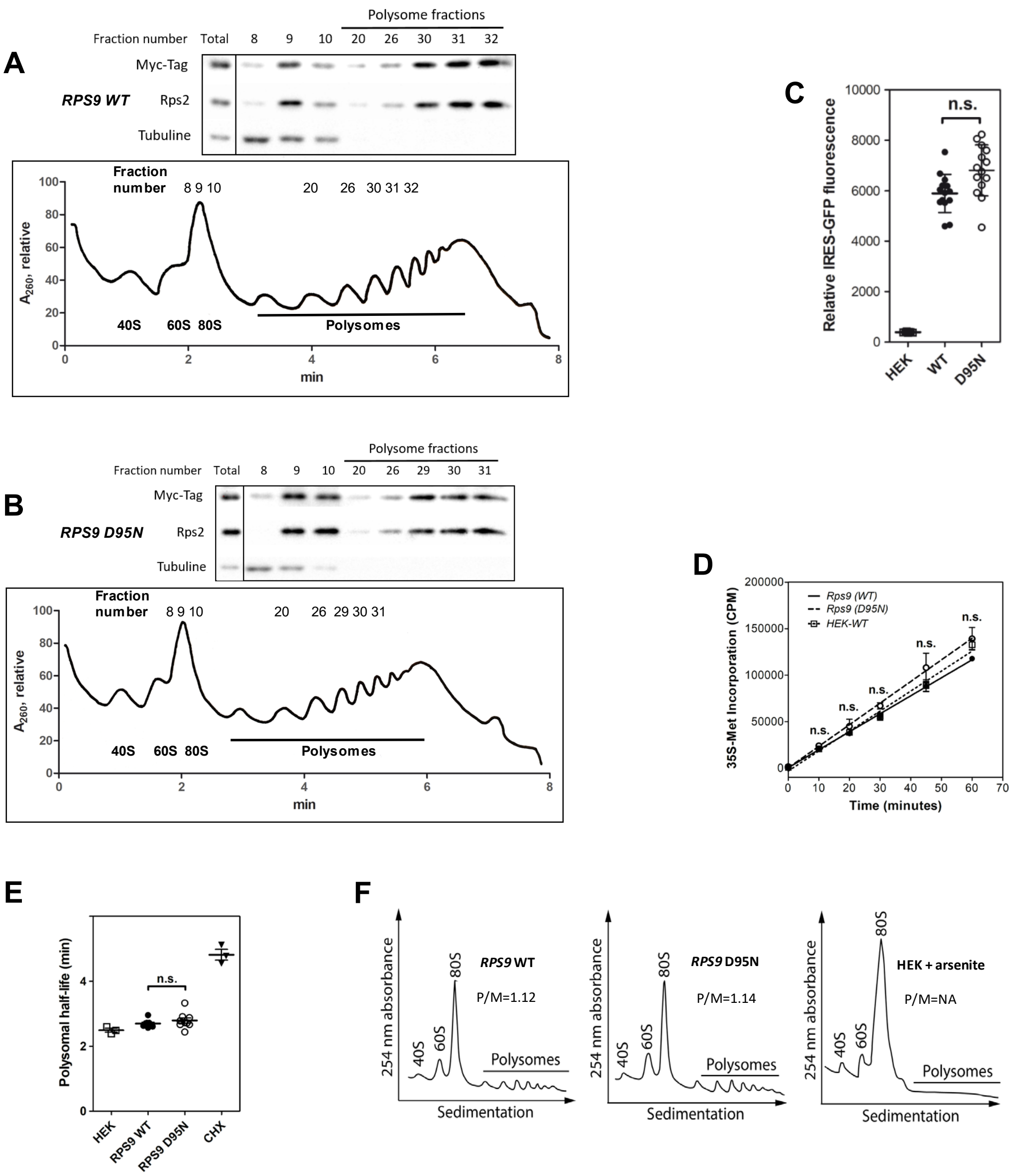

G

	WT			D95N		
	Total	SN	Pellet	Total	SN	Pellet
Fluc counts	199196	197937	240923	339437	206986	663476

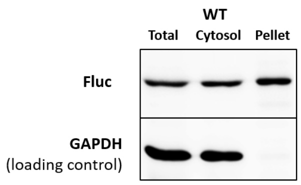

S/T	0.99	0.61
P/T	1.21	1.95
P/S	1.22	3.21

Average (N=3)	WT	D95N
S/T	0.97	0.65
P/T	1.31	1.72
P/S	1.34	2.69
Aggregation D95N/WT	2.0	

p = 0.011

Fig. S3. Analysis of transgene protein expression and translation characteristics in Rps9 transfected cells. (**A, B**) Western blot showing localization of myc-tagged RPS9 transgenic protein (**A** = RPS9 WT; **B** = RPS9 D95N) in ribosome and polysome fractions. Lysates of whole cell and fractions from linear 5-50% sucrose density gradient were analyzed, fractions used for analysis are marked. Myc-tag antibodies were used for detection of transgenic RPS9, RPS2 antibodies were used as control. Tubulin antibodies were used as a control for cytosolic protein. (**C**) *Rps9* transgene expression monitored by IRES-GFP fusion fluorescence as determined by FACS. (N=6 clones each for RPS9 WT and D95N; ± SEM; technical duplicates are shown). (**D**) *In vivo* protein synthesis determined by radiolabelled methionine (^{35}S-Met) incorporation (N=3 clones each for RPS9 WT and D95N; ± SEM). (**E**) Polysome half-life as derived from polysome run-off assays (N=4 clones each for RPS9 WT and D95N; ± SEM; technical duplicates are shown). Treatment with elongation inhibitor cyclohexamide (CHX) used as positive control. (**F**) Polysome profiling of RPS9 WT and RPS9 D95N ribosomes; 0.5 mM arsenite-treated HEK WT cells were used as a control. The polysome to monosome (P/M) ratios were calculated using the area under the curve of the polysomes and the 80S monosome peaks. Representative figures are shown (N=3 clones each for RPS9 WT and D95N). (**G**) Representative WB showing luciferase-based aggregation assay and its quantification.

Fig. S4.

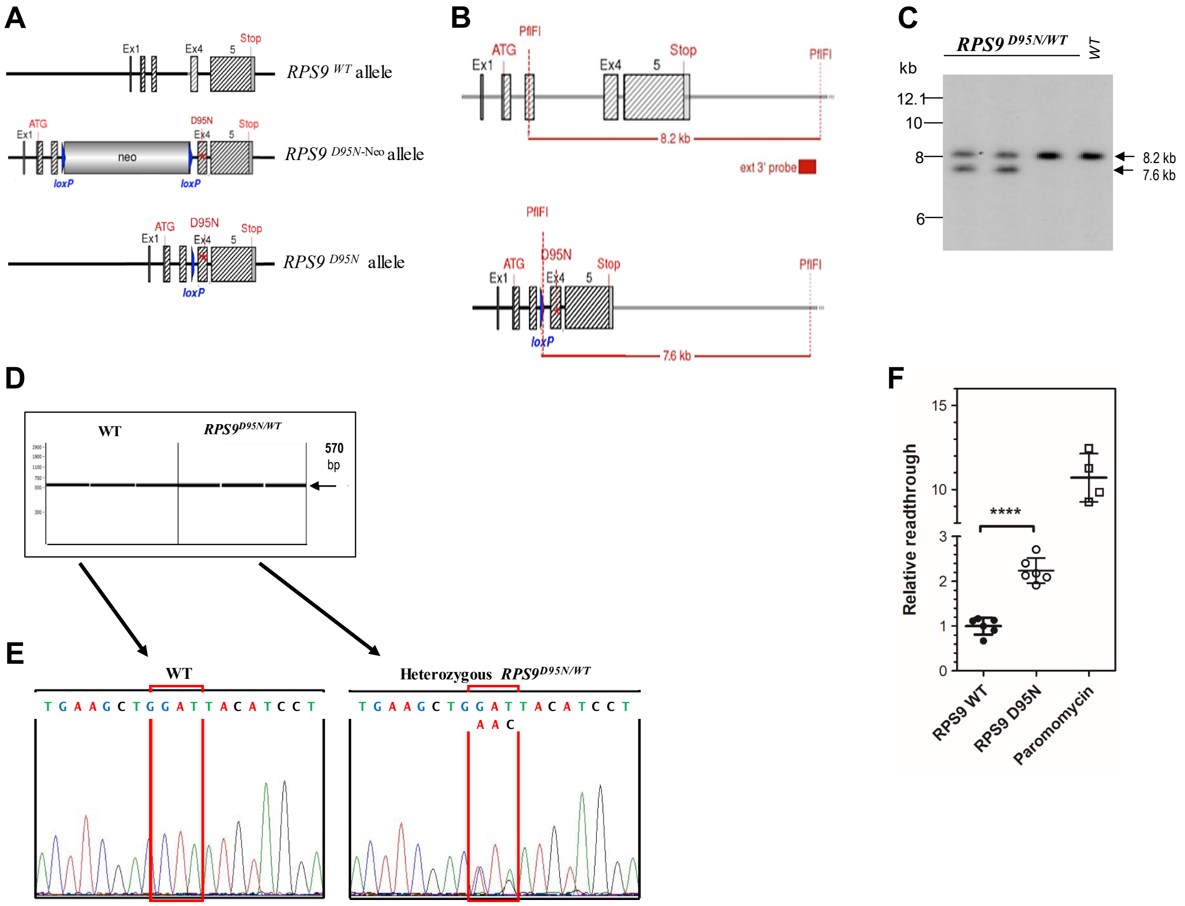

Fig. S4. Generation of *Rps9*$^{\text{D95N/WT}}$ mutant mice and mistranslation in Rps9 D95N mice. (**A**) Schematic representation of the Rps9 alleles. Diagrams are not depicted to scale. Hatched rectangles represent coding sequences, black rectangles indicate non-coding exons portion, solid lines represent chromosome sequences. *lox*P sites are represented by blue triangles. The neomycin cassette is represented by a grey rectangle. (**B**) Southern blot strategy for detection of RPS9WT and RPS9D95N alleles. Restriction sites and external 3' probe used for Southern blot are indicated. (**C**) Representative example of the Southern blot analysis. The genomic DNA of three D95N mutant mice was compared to WT DNA (WT). PflFI digested DNA was blotted on nylon membrane and hybridised with the external 3' probe indicated. (**D**) Representative RT-PCR result on *Rps9*$^{\text{D95N/WT}}$ and WT mice (brain). (**E**) Representative sequencing results of corresponding RT-PCR amplicons. (**F**) Mistranslation assay using Dual Luciferase System. Stop codon read-through of *in vitro* translating ribosomes extracted from liver of the D95N and WT mice (number of animals: WT 3M/3F, D95N 3M/3F); paromomycin (5 µM) is used as a positive control; ****$p<0.0001$. Compared to *Rps9* D95N transfected cells (see Fig. 1C) lower levels of stop codon read-through are observed in liver ribosomes from heterozygous *Rps9* D95N animals measured in *in vitro* cell free translation assays, reflecting different assay conditions and the different ratio of mutant to WT ribosomes (50:50 for heterozygous transgenic animals, >90% mutant ribosomes in transfected cells).

Fig. S5.

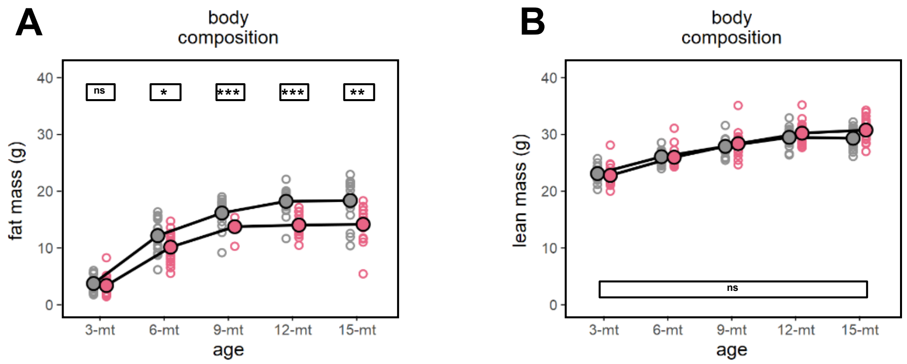

Fig. S5. Body composition. Red = D95N mice, grey = WT. Graphs show untransformed mean, SE and individual data points. Genotype effect overall or split post-hoc by age (white boxes): ***$p<0.001$, **$p<0.01$, *$p<0.05$, ~$p<0.1$, ns $p\geq0.1$. (**A**) Repeated estimates of total body fat in male mice (age $F_{1,138}=305.9$ $p<0.0001$ $\eta^2=0.62$, genotype $F_{1,33}=14.20$ $p=0.0006$ $\eta^2=0.27$, genotype x age $F_{1,138}=3.408$ $p=0.0670$ $\eta^2=0.01$, Box-Cox λ 0.500 @ 0.54900). (**B**) Repeated estimates of total lean mass in male mice (age $F_{1,138}=624.2$ $p<0.0001$ $\eta^2=0.65$, genotype $F_{1,33}=0.592$ ns, genotype x age $F_{1,138}=9.183$ $p=0.0029$ $\eta^2=0.02$). Number of animals (**A, B**): WT 18M, D95N 18M.

Fig. S6.

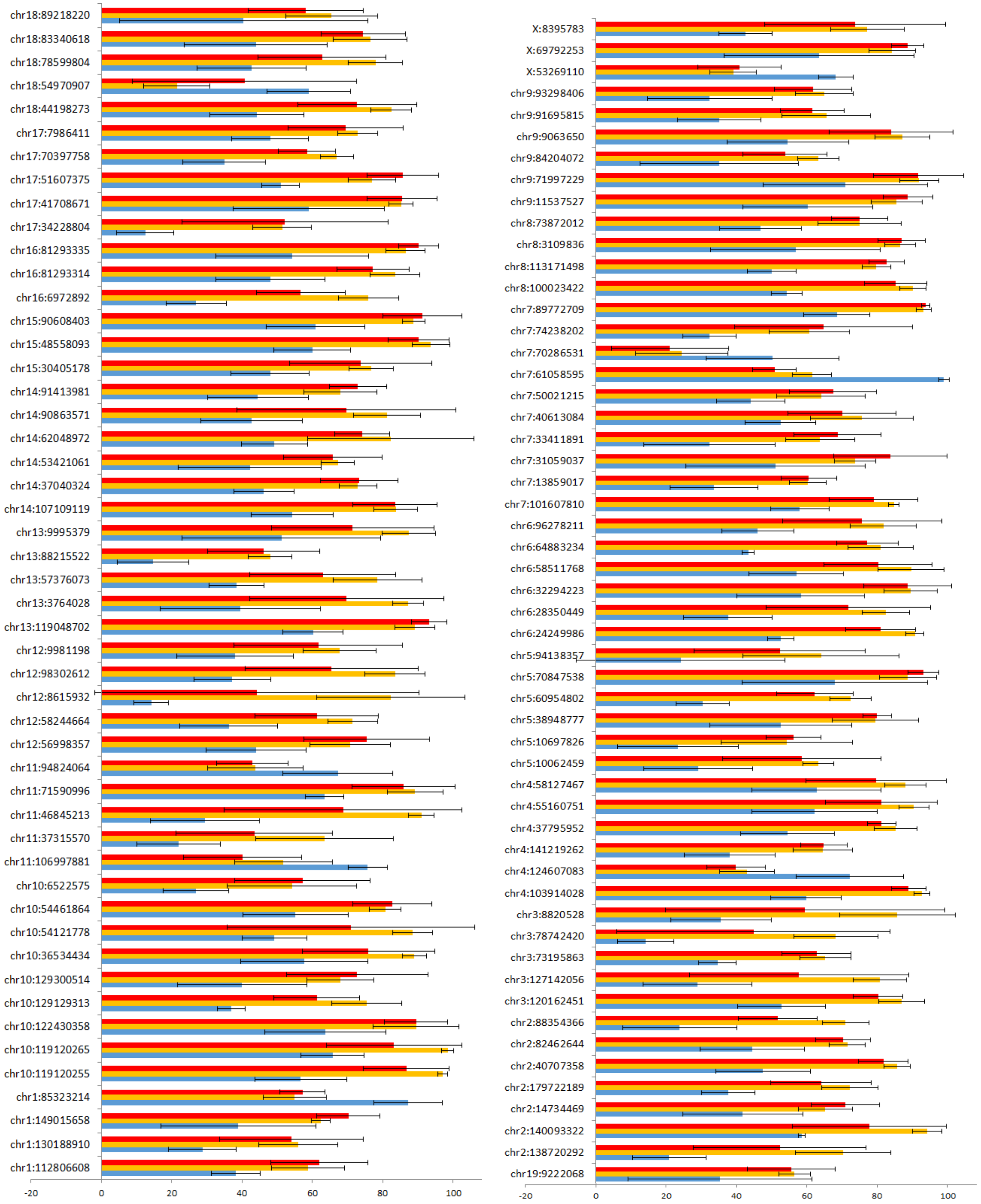

Fig. S6. Methylation levels (in %) of 104 age-related CpG sites. 3 months-old WT (blue), 3 months-old mutant (red), and 18 months-old WT (orange) female mice. N=4 for each group; the error bars show standard deviation.

Table S1. Blood cells count analysis in 18 months female mice – *Rps9* D95N mutants *versus* littermate controls

Genotype	WBC (10^3/uL)	RBC (10^6/uL)	HGB (g/dL)	HCT (%)	MCV (fL)	MCH (pg)	NEUT# (10^3/uL)	LYMPH# (10^3/uL)	MONO# (10^3/uL)	EO# (10^3/uL)	BASO# (10^3/uL)	LYMPH% (%)	MONO% (%)	EO% (%)
RPS9 $^{WT/WT}$	5.62	9.6	13.3	47.6	49.6	13.9	0.87	4.06	0.42	0.27	0	72.2	7.5	4.8
RPS9 $^{WT/WT}$	5.76	9.96	13.9	49.3	49.5	14	1.03	4.23	0.36	0.13	0.01	73.4	6.3	2.3
RPS9 $^{WT/WT}$	6.26	9.93	14.4	50.5	50.9	14.5	0.64	4.63	0.85	0.11	0.03	74	13.6	1.8
RPS9 $^{WT/WT}$	6.09	9.98	14.2	50	50.1	14.2	1.25	4.21	0.52	0.1	0.01	69.1	8.5	1.6
RPS9 $^{WT/WT}$	4.78	9.68	14.1	49.1	50.7	14.6	1.13	3.09	0.44	0.11	0.01	64.6	9.2	2.3
Average	5.7	9.83	13.98	49.30	50.16	14.24	0.98	4.04	0.52	0.14	0.01	70.66	9.02	2.56
St. Dev	0.57	0.18	0.42	1.10	0.63	0.30	0.24	0.57	0.19	0.07	0.01	3.88	2.78	1.29
RPS9 $^{WT/D95N}$	5.13	9	12	42.5	47.2	13.3	1.41	2.51	1.04	0.16	0.01	48.9	20.3	3.1
RPS9 $^{WT/D95N}$	3.6	9.66	13.6	46.7	48.3	14.1	1.01	1.78	0.66	0.14	0.01	49.4	18.3	3.9
RPS9 $^{WT/D95N}$	3.81	9.29	12.7	45.2	48.7	13.7	0.47	2.48	0.49	0.36	0.01	65.1	12.9	9.4
RPS9 $^{WT/D95N}$	3.69	9.29	13.3	45	48.4	14.3	0.67	2.49	0.38	0.14	0.01	67.5	10.3	3.8
Average	4.1	9.31	12.90	44.85	48.15	13.85	0.89	2.32	0.64	0.20	0.01	57.73	15.45	5.05
St. Dev	0.72	0.27	0.71	1.74	0.66	0.44	0.41	0.36	0.29	0.11	0.00	9.95	4.64	2.92
**T-test**	**0.011**	**0.021**	**0.046**	**0.007**	**0.003**	0.193	0.703	**0.001**	0.493	0.410	0.704	**0.075**	**0.062**	0.189

Significant (p<0.05-0.075) changes are marked in **bold**.

Microsoft Excel files:

Table S2. Liver age-related methylation sites 18 m WT vs 3 m WT

Table S3. Liver mutation-associated methylation sites 3 m mut vs 3 m WT

Table S4. Liver cross-comparison of age- and mutation-related methylation sites
